# Is Graphene Always Effective in Reinforcing Composites? The Case of Highly Graphene-Modified Thermoplastic Nanofibers and Their Unfortunate Application in CFRP Laminates

**DOI:** 10.3390/polym14245565

**Published:** 2022-12-19

**Authors:** Emanuele Maccaferri, Laura Mazzocchetti, Tiziana Benelli, Jacopo Ortolani, Tommaso Maria Brugo, Andrea Zucchelli, Loris Giorgini

**Affiliations:** 1 Department of Industrial Chemistry “Toso Montanari”, University of Bologna, Viale Risorgimento 4, 40136 Bologna, Italy; 2 National Interuniversity Consortium of Materials Science and Technology (INSTM), 50121 Florence, Italy; 3 Interdepartmental Center for Industrial Research on Advanced Applications in Mechanical Engineering and Materials Technology (CIRI-MAM), University of Bologna, Viale Risorgimento 2, 40136 Bologna, Italy; 4 Department of Industrial Engineering, University of Bologna, Viale Risorgimento 2, 40136 Bologna, Italy

**Keywords:** polyethylene oxide, polycaprolactone, polyamide, graphene, thermal properties, mechanical properties, delamination, interlaminar fracture toughness, epoxy, carbon fiber composite

## Abstract

Graphene (G) can effectively enhance polymers’ and polymer composites’ electric, thermal, and mechanical properties. Nanofibrous mats have been demonstrated to significantly increase the interlaminar fracture toughness of composite laminates, hindering delamination and, consequently, making such materials safer and more sustainable thanks to increased service life. In the present paper, poly(ethylene oxide) (PEO), polycaprolactone (PCL), and Nylon 66 nanofibers, plain or reinforced with G, were integrated into epoxy-matrix Carbon Fiber Reinforced Polymers (CFRPs) to evaluate the effect of polymers and polymers + G on the laminate mechanical properties. The main aim of this work is to compare the reinforcing action of the different nanofibers (polyether, polyester, and polyamide) and to disclose the effect of G addition. The polymers were chosen considering their thermal properties and, consequently, their mechanism of action against delamination. PEO and PCL, displaying a low melting temperature, melt, and mix during the curing cycle, act via matrix toughening; in this context, they are also used as tools to deploy G specifically in the interlaminar region when melting and mixing with epoxy resin. The high extent of modification stems from an attempt to deploy it in the interlaminar layer, thus diluting further in the resin. In contrast, Nylon 66 does not melt and maintain the nanostructure, allowing laminate toughening via nanofiber bridging. The flexural properties of the nanomodifed CFRPs were determined via a three-point bending (3PB) test, while delamination behavior in Mode I and Mode II was carried out using Double Cantilever Beam (DCB) and End-Notched Flexture (ENF) tests, respectively. The lack of a positive contribution of G in this context is an interesting point to raise in the field of nanoreinforced CFRP.

## 1. Introduction

Today, the availability of high-performance and light materials is crucial to meeting the demand for increasingly sustainable materials, reducing fuel consumption and CO_2_ emissions, and lowering energy requirements for their production with respect to common structural materials (metals) [1,2]. In this frame, composite materials with their light weight, high strength, excellent mechanical properties, corrosion resistance, and fatigue resistance appear to be the most promising candidates for substituting traditional materials as metals and ceramics, also helping in terms of weathering resistance [3]. In most cases, thermosetting composite laminates, such as Carbon Fiber Reinforced Polymers (CFRPs), are ideal for metal replacement. However, they have some drawbacks that limit their usage; among the others, the main ones are: (i) low mechanical resistance at relatively high temperatures [4], (ii) flammability [5], and (iii) an intrinsic vulnerability toward delamination due to their predominantly laminar structure [6]. The first issue is intrinsically related to the chemical structure of the CFRPs, and while it can be addressed with the choice of a convenient polymeric matrix, it cannot be completely overcome. The fire-related question can be mitigated by adding flame-retardant and/or flame-resistant additives to the matrix [7,8,9] or applying surface localized treatments in order to avoid bulk modification [10], and the latter by increasing the interlaminar fracture toughness [11,12,13], that is, the energy required for crack propagation. While bulk matrix toughening is possible, similar to the flame retardancy approach, it is undesirable due to the laminate weight increase and worsening of the overall thermomechanical properties [14,15]. In contrast, localized toughening at the interface region allows for the best balance between improved delamination resistance and the retention of fundamental thermal and mechanical properties [16,17].

Nanofibers are versatile nanomaterials used in a wide range of applications, including filtration [18,19], healthcare, and tissue engineering [20,21,22], sensing [23,24,25], and catalysis [26,27,28]. When nanofibrous membranes are integrated into composite laminates, they can effectively hinder delamination. Common nanofibers include thermoplastic semicrystalline polymers, such as polyamides (Nylon 6 and 66), poly(ε-caprolactone) (PCL), and poly(vinylidene fluoride) (PVDF) [29,30,31,32]. Recently, the well-known poly(ethylene oxide) (PEO), commonly used in health and biomedical applications, has been proposed as a suitable toughener (in nanofibrous form) for epoxy-based CFRP laminates [33]. Moreover, rubbery nanofibers have recently been proposed as interlaminar modifiers for hindering delamination: the high toughening ability delivered by the elastomer significantly raises interlaminar fracture toughness up to +480% [34], even with a negligible lowering of the laminate glass transition temperature (*T*_g_) [35]. Thermoplastics, even modified bulk thermoplastics, have already been reported in the literature for modifying mechanical performance [36].

Among nanomaterials, graphene (G) has also attracted great expectations since its discovery in 2004 [37]. Indeed, thanks to its outstanding mechanical properties, and high thermal and electrical conductivity, particular attention and research have been devoted not only to graphene’s own properties but also to “transferring” these properties to other materials by modifying them with it. Graphene oxide (GO) and reduced graphene oxide (rGO), which belong to Graphene and Related Materials (GRMs), can also be used to impart new properties to polymeric substrates [38,39]. In particular, the attempt to deploy G in specific regions of a composite might help enhance local properties such as electrical conductivity. This feature could also be helpful in the presence of nanostructured molecular actuators (nano-machines and similar items) to power and retrieve signals generated in situ. Polymers are some ideal substrates for GRM addition to obtain peculiar properties useful in several fields, ranging from electronics to filtration and from catalysis to biomedical engineering [40,41,42].

The small dimensions of GRMs allow them to nanomodify even micro- and nanofibers, enhancing thermal [43] and electrical [44] conductivity, improving mechanical and thermomechanical performance [45,46], and adding other peculiar properties. Applications include sensors [47,48,49], electromagnetic shielded materials [50], supercapacitors [51], tissue engineering [52], photocatalysis [53], fuel cells [54], filtration [55], CO_2_ capture [56], wastewater treatment [57,58], and oil/water separation [59].

G was already used to modify the nanofibers. However, it is worth pointing out that the literature data on the mechanical properties of graphene-reinforced nanofibers are poor. In some cases, an improvement in the mat’s mechanical properties is reported, while in others, degradation seems to occur, mainly depending on the modification extension [45,60,61,62,63].

While the integration of polymeric nanofibers for hindering delamination is well established and documented [29,64], the use of graphene-reinforced nanofibers is almost undiscovered in this field and needs to be investigated. Other nano-hybrid approaches, meaning a twofold nanostructured modification based on two concomitant properties both stemming from nanostructured materials, such as nanocoated nanofibers, were recently implemented for CFRPs modification [65], with significant improvements in thermal and mechanical performance. Thus, combining both nanomaterial types (GRMs and nanofibers) may be synergistically beneficial in contrasting composite delamination and could also represent an alternative way to place GRMs in specific regions using nanofibers as transfer media. Indeed, graphene transfer within fiber-reinforced polymers is still an open point, since the presence of fibrous systems acts as sieving toward GRMs, blocking the flow of this additive throughout the mass of the composite. Hence, a simple and easy way to deploy GRMs, and possibly limiting them to selected regions of interest for modification would be attractive from an industrial point of view.

The present work aims to evaluate the interlaminar fracture toughness of CFRP laminates modified with graphene-reinforced nanofibers using three different thermoplastic polymers as substrates: PEO, PCL, and Nylon 66. These polymers display essential differences, as well as some similarities. PCL and PEO have comparable thermal properties (low glass transition temperatures, *T*_g_s, and low melting temperatures, *T*_m_s), allowing their mixing with the surrounding matrix during laminate curing; however, owing to the different chemistry, their relative affinity in the epoxy could be substantially different. In this way, upon dissolution of nanofibrous morphology, G can be released locally, possibly modifying the region where nanofibers are deployed. In this context, nanofibers will be loaded with an extremely high concentration of G in order to significantly reinforce the surrounding epoxy region. In contrast, polyamide has a higher *T*_m_ that prevents its melting and mixing with the resin during curing, enabling a different reinforcing mechanism at the laminate interface.

## 2. Materials and Methods

### 2.1. Materials

Poly(ethylene oxide) (PEO), M_w_ 100,000 g/mol, and poly(ε-caprolactone) (PCL), M_w_ 70,000–90,000 g/mol, were purchased from Sigma-Aldrich (St. Louis, MO, USA). Nylon 66 (Zytel E53 NC010) was kindly provided by DuPont (Wilmington, DE, USA). Chloroform (CHCl_3_), acetone, *N*,*N*-dimethylformamide (DMF), and formic acid were purchased from Sigma-Aldrich. Polymers and solvents were used without any preliminary treatment.

Plain weave carbon fabric (200 g/m^2^) in epoxy matrix prepreg (GG204P IMP503Z-HT) for composite lamination was supplied by G. Angeloni s.r.l. (Venezia, Italy).

### 2.2. Preparation of Solutions, Electrospinning Process, and Mats’ Characterization

Polymeric solutions without G were prepared by dissolving the right amount of polymer into a preformed solvent system, and then they were stirred until forming a homogeneous solution. The mixture was heated to speed up the polymer dissolution (maximum 60 °C).

The solutions containing G were prepared with the same polymeric concentration and solvent system as the G-free ones. However, before adding the polymer, the G dispersion underwent sonication. After a coarse G dispersion in a sonication bath (model AC 14, Uniset, Rochester, NY, USA), a more vigorous one was carried out using a tip sonicator (model VCX 750, Sonics, 750 W, microtip diameter 3 mm), as follows: amplitude of 30%, on-off cycles of 5-1 s, 45 min of actual sonication). After that, half of the polymer amount was added to the G dispersion and then stirred at 60 °C until its dissolution. Then, the low-concentrated solution underwent further sonication using the same parameters, except for the amplitude, which was raised to 38%. Finally, the last polymer fraction was added, stirred, and sonicated, as described for the first polymer fraction addition.

Table 1 lists the prepared solutions to be processed via electrospinning.

**Scheme 1:**
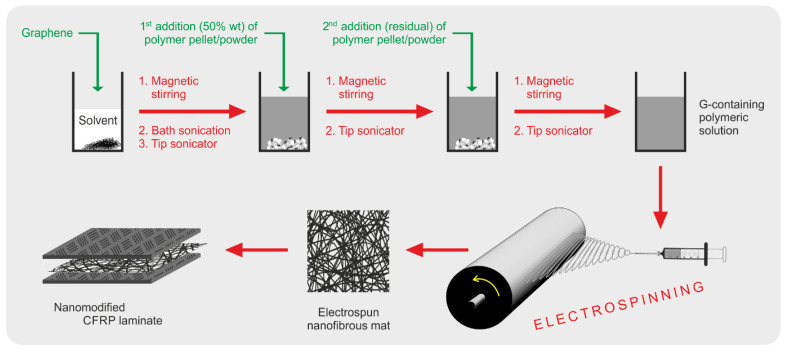
Flow chart representing the steps adopted for the preparation of G-modified nanofibrous mats to be interleaved into CFRP laminates.

Scheme 1 illustrates the steps for dispersing G and preparing G-containing solutions to be electrospun to produce G-nanomodified mats to be interleaved into CFRP laminates.

The nanofibrous mats were produced using an electrospinning machine (Lab Unit, Spinbow s.r.l., Bologna, Italy) equipped with four 5 mL syringes joined via Teflon tubing to translate needles (length 55 mm, internal diameter 0.84 mm). A rotating drum (tangential speed of 0.39 m/s) covered with polyethylene-coated paper was used as a collector.

The electrospinning process was conducted in an air-conditioned room, with 22–23 °C and relative humidity (RH) ranging from 22 to 25%. The selected process parameters and the nanofiber’s diameter evaluation are reported in Table 2.

Mats have final dimensions of 20 × 40 cm; the electrospinning process was carried out until reaching a mat thickness of 40 ± 4 μm, measured with an analog indicator (Borletti, Italy), under 360 g/m^2^ pressure on five different mat regions.

The nanofibers’ morphology was evaluated by scanning electron microscopy (Phenom ProX, ThermoFisher Scientific, MA, USA), recording the images at 10 kV. All analyzed surfaces were previously gold-coated using a Quorum SC7626 sputter coater (180 s, 18 mA) to make them conductive. Average diameter values were calculated from at least 100 measurements, manually done on single nanofibers using the Photoshop measurement tool.

The thermal properties of the nanofibrous mats were evaluated via differential scanning calorimetry (DSC, model Q2000 equipped with an RCS cooling system, TA Instruments—Division of Waters, DE, USA). Samples of 6–8 mg were heated/cooled at 20 °C/min under a nitrogen atmosphere. The degree of crystallinity (*χ_c_*) was calculated according to the well-known Equation (1):

(1) $$\chi_{c}\;\left(\%\right)=\frac{\Delta H_{m}^{exp}}{\Delta H_{m}^{100\%\;cryst}}\cdot 100$$

where $\Delta H_{m}^{exp}$ is the experimental melting enthalpy and $\Delta H_{m}^{100\%\;cryst}$ is the melting enthalpy of a theoretical 100% crystalline polymer. For the *χ_c_* calculation, the following $\Delta H_{m}^{100\%\;cryst}$ were considered: 203−205 J/g for PEO [66,67]; 139.5 J/g for PCL [68]; 196 J/g for Nylon 66 [69]. In the case of G-loaded nanofibers, the $\Delta H_{m}^{exp}$ has been normalized with respect to the actual polymer fraction.

### 2.3. Production and Characterization of CFRPs

**Figure 1:**
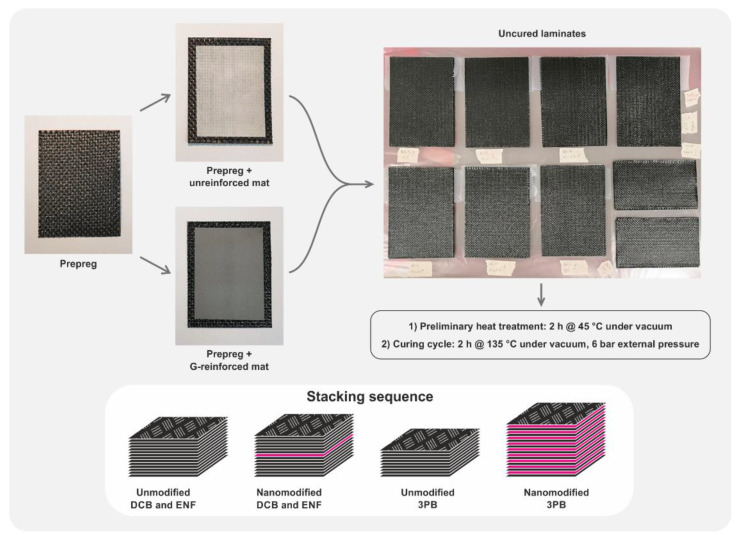
Production of nanomodified laminates and stacking sequence for DCB, ENF, and 3PB specimens.

Composite laminates were prepared via hand lay-up, interleaving the nanofibrous mats where necessary, as depicted in Figure 1, in an air-conditioned room (22–23 °C, 22–25% RH). Before laminate curing in an autoclave (2 h at 135 °C, under vacuum, 6 bar external pressure, heating/cooling ramp 2 °C/min), a preliminary mild heat treatment (2 h at 45 °C under vacuum) was applied for better impregnation of nanofibers.

Specimens for the interlaminar fracture toughness evaluation via Double Cantilever Beam (DCB) and End-Notched Flexure (ENF) tests were fabricated stacking 14 prepreg plies, interleaving a nanofibrous mat in the central interface, using a Teflon film as crack trigger. Specimens for the three-point bending (3PB) test were produced by stacking 10 prepreg plies, and the nanofibrous mat was inserted between all interfaces. In addition to the nanomodified specimens, unmodified ones were also produced for the sake of comparison. Details of the DCB, ENF, and 3PB specimens are listed in Table 3.

Mechanical tests were carried out using a universal testing machine (Remet TC-10, Bologna, Italy) equipped with a 100 N load cell for DCB tests and a 1 kN load cell for DCB and ENF tests.

DCB and ENF tests were carried out at 3.0 and 2.0 mm/min crosshead separation rates, respectively. The energy release rate for Mode I loading (G_I_, in J/m^2^), both at the initial and propagation stages (G_I,*C*_ and G_I,*R*_, respectively), was evaluated using Equation (2), according to ASTM D5528-01 [70]:

(2) $$G_{I}=\frac{3P\delta }{2ba}$$

where *P* is the load, *δ* is the crosshead displacement, *a* is the crack length, and *b* is the specimen width. The G_I,*R*_ was evaluated considering a crack length range of 47–90 mm.

The energy release rate for Mode II loading (G_II_, in J/m^2^), both at the initial and propagation stages (G_II,*C*_ and G_II,*R*_, respectively), was evaluated using Equation (3), according to BS EN 6034:2015 [71]:

(3) $$G_{\mathrm{II}}=\frac{9P\delta a^{2}}{2b\left(\frac{1}{4}L^{3}+3a^{3}\right)}$$

where *L* is the span length between supports. ENF tests were carried out with a 100 mm span (*L*) between supports, and the specimen was placed in a 3-point bending geometry as follows: 50 mm specimen half-span (*L*/2) and 30 mm delamination length (*a*_0_). The G_II,*R*_ was evaluated considering a crack length range of 31–43 mm.

3PB tests were carried out setting a span of 85 mm (span-to-specimen thickness ratio 32:1) and with a 2.0 mm/min crosshead separation rate, according to ASTM D790.

For each sample/test combination, three repetitions were run.

After the DCB tests, delamination surfaces for investigating the matrix behavior were evaluated via SEM microscopy (Phenom ProX, ThermoFisher Scientific, Waltham, MA, USA).

## 3. Results and Discussion

### 3.1. Morphological Characterization of Nanofibrous Mats

Polymeric nanofibers containing G were prepared by suspending G in solvent systems suitable for polymer solubilization and subsequently adding the polymer stepwise (Table 1). Analogous solutions lacking G were also produced for the sake of comparison. All the solutions were then processed by electrospinning, adjusting the operational parameters to obtain nanofiber mats (Table 2). As previously stated, the G load in the nanofibers is up to 5% wt, an amount of graphenic derivative that is extremely above the average typical reinforcing range. This is because the G content in the nanofibers that melt upon curing after insertion within CFRP laminae is expected to be deployed within the epoxy resin and act as reinforcement of the wider epoxy volume. All electrospun mats were analyzed via SEM to evaluate their morphology. While plain nanofibers showed smooth surfaces and a cylindrical shape, the nano-reinforced ones displayed protrusions due to the high concentration of G nanoplatelets, whose dimensions did not allow for their complete accommodation within the nanofiber. This fact was previously observed when loading a high G amount [45]. However, it is worth noting that G is located not only in the protrusions but also along the nanofiber, as suggested by the not “perfectly” cylindrical shape and the less smooth fiber surface.

**Figure 2:**
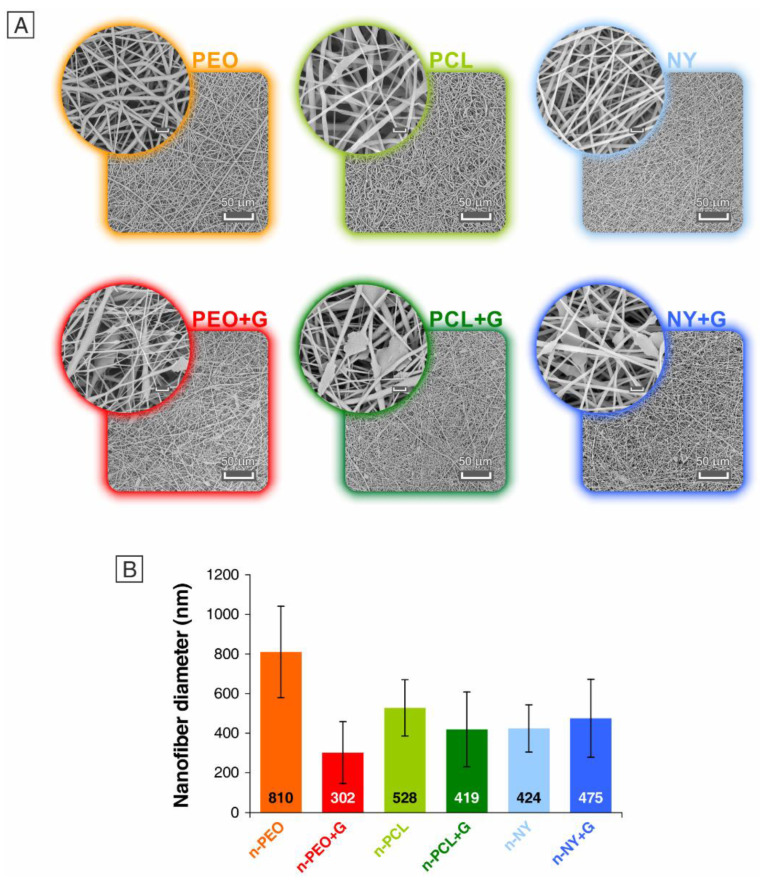
(**A**) SEM images of PEO, PCL, and Nylon 66 nanofibers, unreinforced, and G-reinforced ones. Scale bar of enlarged images: 4 μm. (**B**) Histograms of the nanofibers’ mean diameters.

G surely modifies the electrical conductivity of the polymeric solutions, thus affecting their interaction with the applied electrostatic field during electrospinning processing. Generally, if the other process parameters are maintained unchanged, such as flow rate, voltage, and needle-to-collector distance, the net effect is a diameter reduction. However, especially at such a high G percentage, the solution viscosity may also increase, making the polymeric jet less prone to be stretched by the electrostatic field. The resulting overall effect on the fiber diameter derives from these two contrasting phenomena [45]. Here, diameter reduction was the predominant effect for G-modified PEO nanofibers, whose diameter was less than halved (302 vs. 810 nm, Figure 2 and Table 2). Instead, G did not significantly affect the mean diameter of the PCL and Nylon 66 nanofibers.

### 3.2. Thermal Characterization of Nanofibrous Mats

**Figure 3:**
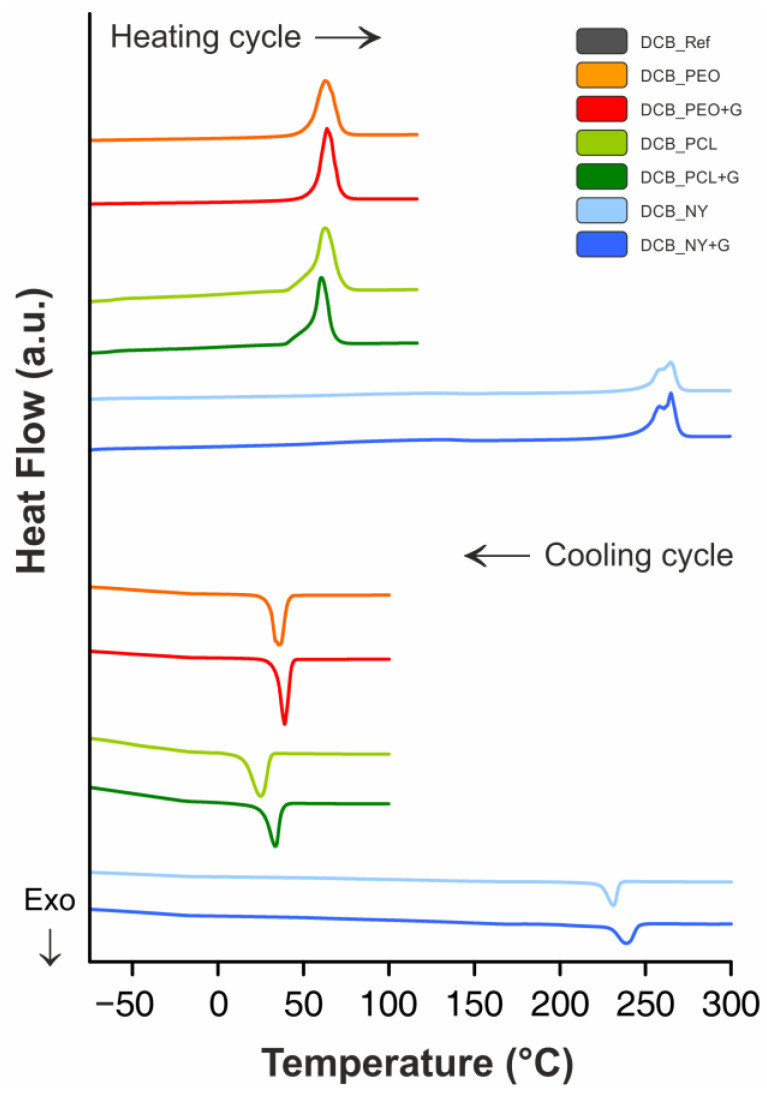
Thermal behavior of PEO, PCL, and Nylon 66 assessed via DSC analysis. Top thermograms: heating cycle; bottom thermograms: cooling cycle.

In the present comparative work, the selected polymers displayed similarities and differences among them. The polyether PEO and the polyester PCL had almost the same thermal properties, as assessed via DSC analysis (Table 4 and Figure 3), but different chemical structures and, in turn, different interactions with the epoxy matrix. This polymer pair can act against delamination exclusively via the matrix toughening mechanism; that is, the hosting epoxy matrix becomes (locally) toughened upon polymer melting (*T*_m_ ≈ 60 °C) and mixing with the resin during the curing cycle, requiring a higher energy input for crack propagation. The polyamide, however, cannot melt and mix during composite curing due to its melting temperature (*T*_m_ = 265 °C), which is higher than the typical temperatures (120–140 °C) set for curing high-performance epoxy laminates. In this case, the 3D nanofibrous network is still present in the cured laminate, enabling composite toughening via so-called nanofiber bridging. Additionally, the net effect is increased interlaminar fracture toughness.

It was previously demonstrated that carbon nano-reinforcements could affect the thermal behavior of thermoplastics [42]. Here, the graphene addition did not substantially impact the fundamental thermal properties (*T*_g_ and *T*_m_, Table 4 and Figure 3), nor the polymer crystallinity (χ_c_), which was comparable to unreinforced nanofibers. PEO nanofibers represent an exception; the G addition further promoted slight crystallinity development (χ_c_ = 87% instead of 83%). Conversely, a decrease in χ_c_ was previously observed for similar Nylon 66 nanofibers with the same G amount [45]. In the same work, by analyzing the thermal behavior of nanofibers modified with very different G amounts, a threshold was found between 1.5 and 2.0% wt for a change in behavior. Indeed, the crystalline fraction is comparable with the unreinforced nanofibers up to 1.5% wt, while above 2.0% wt, the development of crystallinity is hindered. However, the solvent system used for nanofibers’ electrospinning was different, as were the electrospinning conditions, suggesting that solution and process parameters play a fundamental role in determining the nanofibers’ thermal properties. In all cases, G acts as a nucleating agent, as can be inferred by analyzing the crystallization onset ($T_{c}^{onset}$) and crystallization temperature at peak ($T_{c}^{peak}$); the crystal development is indeed anticipated thanks to the G presence, as already observed [45].

### 3.3. Mechanical Properties of CFRP Laminates

**Figure 4:**
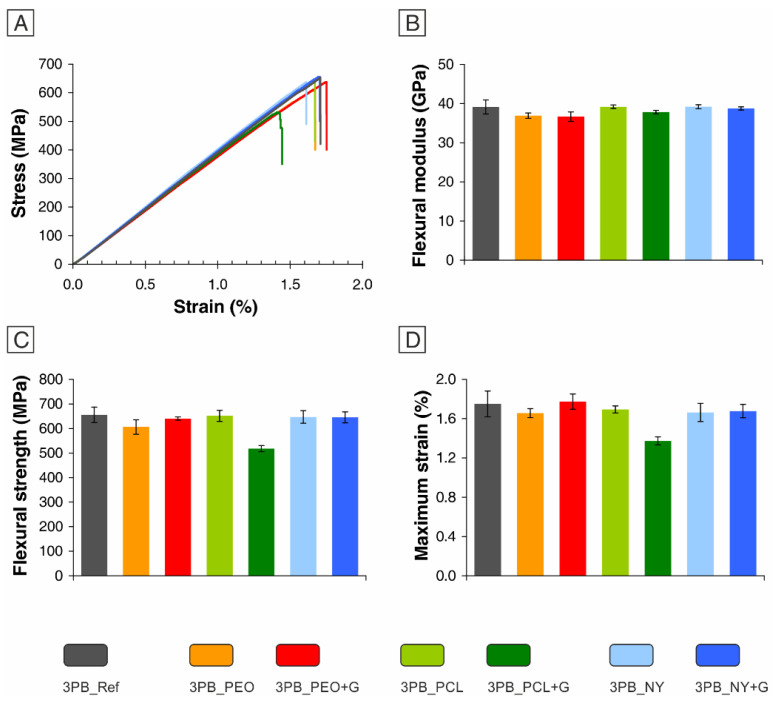
3PB test: (**A**) Stress–strain curves; (**B**) elastic modulus; (**C**) maximum strength; (**D**) maximum strain.

The laminates’ mechanical properties were evaluated using the 3PB test (Figure 4). The original mechanical properties were almost retained by the nanomodified CFRPs. Indeed, the nanomodification did not affect the flexural modulus (Figure 4B), nor the flexural strength (Figure 4C) and maximum strain (Figure 4D), except for properties at break of the 3PB_PCL + G sample, which experienced a reduction (~20%).

### 3.4. Delamination Behavior of CFRP Laminates

**Figure 5:**
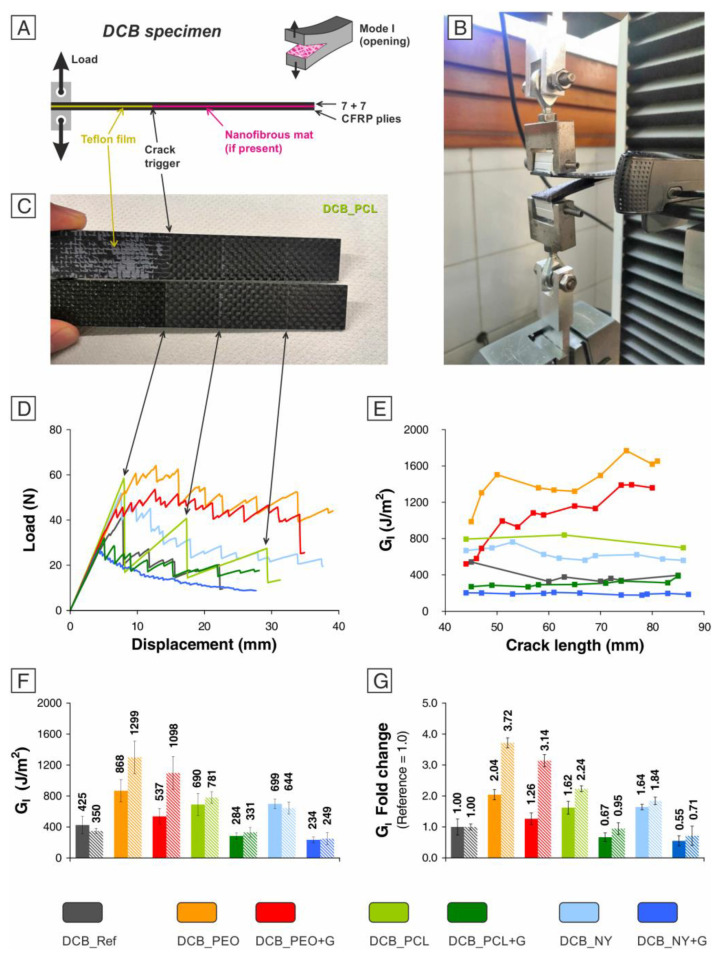
DCB test. (**A**) Sketch of DCB specimen; (**B**) Running DCB test; (**C**) Delaminated specimen (represented as a DCB_PCL specimen clearly displaying the crack advancements); (**D**) load-displacement curves; (**E**) *R*-curves related to the same specimens displayed in (**D**); (**F**) average G_I,C_ and G_I,R_; (**G**) G_I_ fold change (bars are expressed as the relative variation of the value with respect to the reference sample, DCB_Ref, whose value is set as 1.0). In (**F**) and (**G**), solid bars are G_I,C_ values, and dashed bars G_I,R_ values.

Delamination resistance was evaluated in Mode I and Mode II via DCB and ENF tests, respectively. As anticipated, the polymers selected for nanofiber fabrication may lead to different interlaminar reinforcing mechanisms: so-called nanofiber bridging and matrix toughening. In addition to mixing with the surrounding epoxy resin, the low-*T*_m_ PEO and PCL might also promote G spreading within the surrounding epoxy matrix. In contrast, the high-*T*_m_ polyamide cannot transfer G as the other two thermoplastics. In this case, however, the G sheets protruding from the nanofibers may improve the nanofiber–resin interaction thanks to the augmented exposed surface area and a possible anchoring mechanism similar to the action of barbed wire. Table 5 and Figure 5 summarize the DCB test results.

Load-displacement curves (Figure 5D) represent the raw data recorded by the testing machine. They provide an initial “picture” of the materials’ delamination behavior; a curve positioned, on average, higher than that of the unmodified CFRP indicates that the laminate crack occurs at higher loads. Since each peak represents the maximum load endured by the laminate an instant before crack advancement occurrence, its number is related to the “frequency” of crack advancements. Some different behaviors of crack growth can be observed by analyzing the load-displacement curves of the different CFRPs. In particular, crack propagation can be characterized by:

(1) A high average load with frequent drops (DCB_PEO and DCB_PEO + G samples);
(2) A medium average load with frequent drops (DCB_NY sample);
(3) A medium average load with rare drops (DCB_PCL);
(4) A low average load with even rarer drops (DCB_Ref and DCB_PCL + G);
(5) A very low average load with very frequent and low drops (DCB_NY + G).

The curves of both PEO-modified laminates were positioned at significantly higher loads with respect to the unmodified laminate, hinting that crack advancement required higher loads. The *R*-curves (Figure 5E) derived from the G_I_ calculations confirm that the PEO-modified composites require more energy for delamination than the reference material. In particular, the G_I,*C*_ doubles and G_I,*R*_ is 3.7× the reference laminate when PEO nanofibers are integrated (DCB_PEO, Table 5). Adding graphene improved the G_I_ with respect to the unmodified material (DCB_PEO + G, +26% in G_I,*C*_, and +214% in G_I,*R*_), but it did not further enhance the performance of the PEO-only nanofiber-modified CFRP. In fact, graphene lowers G_I_, especially G_I,*C*_. This behavior is general; the other two nanofiber types display a similar trend. While PCL and Nylon 66 unreinforced nanofibers enable a delamination hindering, in line with the literature data [29,34,64,72,73], graphene addition dramatically lowers G_I_. The DCB_NY + G sample displayed the worst performance, showing an almost halved G_I_. This laminate displayed completely different behavior with respect to one of the DCB_NY samples; the load-displacement curve was characterized by frequent and small drops, which may indicate a low adhesion between the nanofibers and the matrix, as already found when polyaramid nanofibers (Nomex) were integrated [35], and/or a lower effective nanofiber bridging.

Regarding the DCB_PCL + G laminate, the graphene addition, again, leads to modifications of both the load-displacement profiles and interlaminar fracture toughness. While the maximum load in propagation was similar to the reference sample, there was a significant drop in the load associated with the first crack advancement, resulting in G_I_ reduction by one-third. As in the case of the DCB_NY + G sample, graphene led to a lowering in the gap between the maximum loads (peaks) and the minimum loads achieved just after crack propagation. Moreover, such behavior is extremely amplified in the case of the DCB_PCL + G laminate. Indeed, the delamination of the DCB_PCL sample occurred with only three crack advancements (still visible on the delamination surfaces shown in Figure 5C), while the laminate with PCL + G nanofibers displayed a number of crack advancements similar to the unmodified laminate.

**Figure 6:**
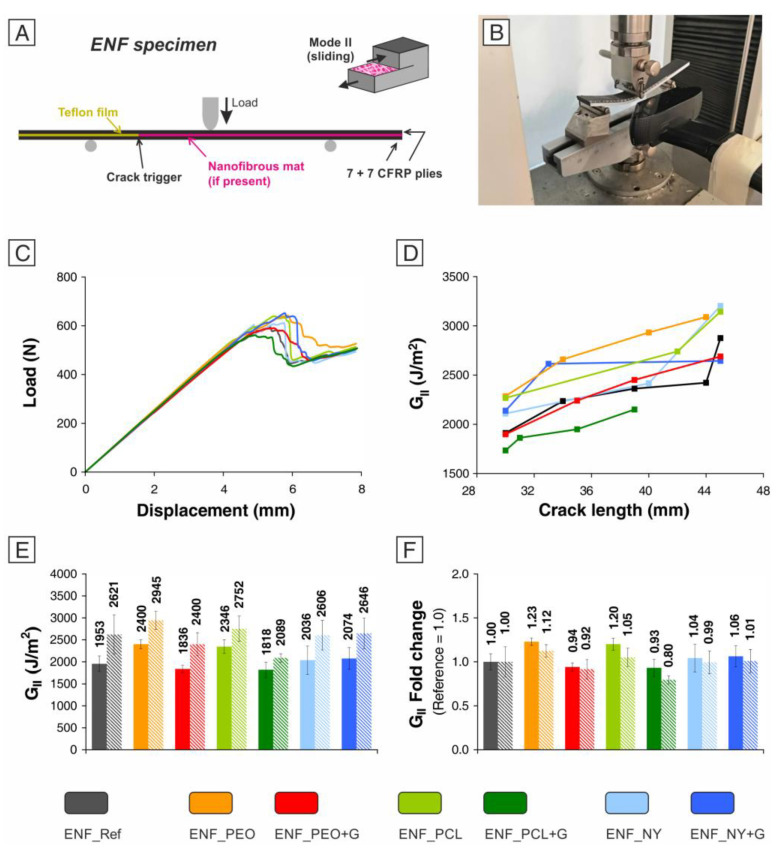
ENF test. (**A**) Sketch of ENF specimen; (**B**) running ENF test; (**C**) load-displacement curves; (**D**) *R*-curves related to the same specimens displayed in (**C**); (**E**) average G_II,C_ and G_II,R_; (**F**) G_II_ fold change (bars are expressed as the relative variation of the value with respect to the reference sample, ENF_Ref, whose value is set as 1.0). In (**E**) and (**F**), solid bars are G_II,C_ values, and dashed bars are G_II,R_ values.

The effect on G_II_ (Figure 6) was more limited than in the case of G_I_. Indeed, the maximum increment achieved is 23% by the ENF_PEO sample, followed by ENF_PCL one (+20%). The other samples behaved as unmodified laminates, or even slightly worse, such as ENF_PEO + G and ENF_PCL + G. The modification with Nylon 66 nanofibers, with and without graphene, did not lead to any G_II_ variation.

**Figure 7:**
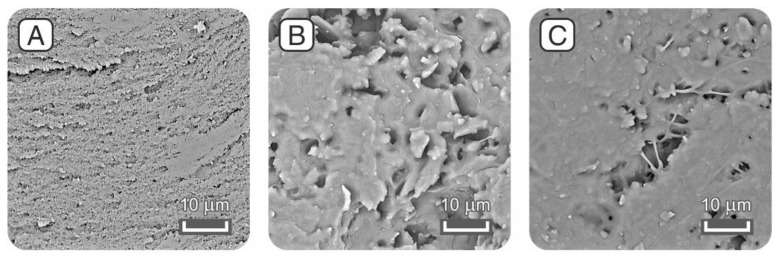
SEM images of delamination surfaces from DCB specimens: (**A**) DCB_PEO + G, (**B**) DCB_PCL + G, and (**C**) DCB_NY + G.

SEM images recorded on DCB delamination surfaces confirmed PEO and PCL melting, in addition to maintaining Nylon 66 nanofibers upon the curing cycle. The aspect of fractured surfaces also supported the ability of both low melting thermoplastics (PEO and PCL) to toughen the epoxy resin, highlighting a rougher surface aspect typical of plastic deformation phenomena occurring during crack propagation. No trace of residual nanofibrous morphology can be retrieved from the SEM investigation. Conversely, NY and NY + G laminates both still display reminiscence of the nanofibrous mat persistence clearly appearing within the plastic resin bulk, supporting the fact that the electrospun membrane is well soaked with the epoxy resin during curing. In none of the analyzed G-containing samples (Figure 7), it is possible to track down the presence of graphenic sheets. This technique, indeed, is not suitable for investigating G dispersion in organic matrices, since there is no significant difference in the elemental composition that could drive measurable phenomena. Moreover, the presence of carbon fibers, which are structurally analogous to graphene in terms of chemical structure, represents an additional drawback when attempting to trace the nanostructured additive in real matrices that are not built on purpose for G detection and analysis. This lack of investigation techniques suitable for the task of graphene tracing still hampers the full comprehension of G delivery within the resin, which might help in fully understanding the present results.

## 4. Conclusions

In the present work, highly G-loaded nanofibers based on different polymers were produced with the aim of using them as reinforcing agents in CFRP laminates. The polymers were chosen considering their thermal properties and, consequently, their mechanism of action against delamination. PEO and PCL, displaying a low melting temperature, melt and mix during the curing cycle; they are used as tools to deploy G specifically in the interlaminar region, with the aim of imparting interlaminar strength via matrix toughening, while Nylon 66 does not melt and maintain the nanostructure, acting via nanofiber bridging. The high extent of modification stems from the attempt to deploy G in the interlaminar layer, which will be diluted further when mixing with the surrounding epoxy resin. In all cases, the flexural properties of the nanomodifed CFRPs, determined via three-point bending (3PB) tests, showed results consistent with the plain CFRP, demonstrating that the nanofibrous approach is not detrimental to overall mechanical performance. While the plain nanofibers made of the same polymers all showed positive results in terms of interlaminar fracture reinforcement, the present unfortunate results demonstrate that the addition of graphene is not relevant, if not even detrimental, in some cases, in terms of interlaminar fracture toughness. It seems that the ability to spread G in specific regions via dissolution of the polymeric nanofibrous carrier during the curing process of the epoxy resin does not contribute further to the potential of hindering delamination. Nor can better results be achieved with the use of G-modified NY nanofibers that, though acting at reinforcing the polymer via bridging action, still do not contribute any further than the plain unmodified fibers to the ability to contrast the delamination phenomenon. It has, however, to be pointed out that thermal and electrical conductivity evaluations are still underway. Indeed, G is known to impart many interesting functionalities, often all at once. Hence, the lack of mechanical performance is not necessarily a fully negative result in light of a more general properties enhancement for overall composite performance.

## Figures and Tables

**Table 1:** Characteristics of PEO, PCL, and Nylon 66 solutions.

Solution	Polymer Type and Concentration	Graphene *	Solvent System
*s*-PEO	PEO 12% wt	-	CHCl_3_/acetone 60:40 wt
*s*-PEO + G	PEO 12% wt	5% wt	CHCl_3_/acetone 60:40 wt
*s*-PCL	PCL 10% wt	-	CHCl_3_/DMF 50:50 wt
*s*-PCL + G	PCL 10% wt	5% wt	CHCl_3_/DMF 50:50 wt
*s*-NY	Nylon 66 13% wt	-	CHCl_3_/formic acid 50:50 wt
*s*-NY + G	Nylon 66 13% wt	5% wt	CHCl_3_/formic acid 50:50 wt

* The graphene percentage is referred to the polymeric fraction only.

**Table 2:** Electrospinning process parameters and nanofiber diameters.

Nanofibrous mat	Electrospun Solution	Flow Rate (mL/h)	Electric Potential (kV)	Distance (cm)	Nanofiber Diameter (nm)
*n*-PEO	*s*-PEO	0.60	19.0	11	810 ± 231
*n*-PEO + G	*s*-PEO + G	0.60	18.0	15	302 ± 156
*n*-PCL	*s*-PCL	0.70	17.0	11	528 ± 142
*n*-PCL + G	*s*-PCL + G	0.60	17.0	11	419 ± 189
*n*-NY	*s*-NY	0.35	23.5	10	424 ± 119
*n*-NY + G	*s*-NY + G	0.30	23.0	8	475 ± 197

**Table 3:** Details of produced DCB, ENF, and 3PB specimens.

Test Type	Specimen Code	Nanofibrous Mat	Specimen Width (mm)	Specimen Length (mm)	Initial Delamination Length (*a*_0_) (mm)	Span (L) (mm)
DCB	DCB_Ref	-	20	130	45	-
	DCB_PEO	*n*-PEO				
	DCB_PEO + G	*n*-PEO + G				
	DCB_PCL	*n*-PCL				
	DCB_PCL + G	*n*-PCL + G				
	DCB_NY	*n*-NY				
	DCB_NY + G	*n*-NY + G				
ENF	ENF_Ref	*-*	20	160	30	-
	ENF_PEO	*n*-PEO				
	ENF _PEO + G	*n*-PEO + G				
	ENF_PCL	*n*-PCL				
	ENF_PCL + G	*n*-PCL + G				
	ENF_NY	*n*-NY				
	ENF_NY + G	*n*-NY + G				
3PB	3PB_Ref	*-*	15	110	-	85
	3PB_PEO	*n*-PEO				
	3PB_PEO + G	*n*-PEO + G				
	3PB_PCL	*n*-PCL				
	3PB_PCL + G	*n*-PCL + G				
	3PB_NY	*n*-NY				
	3PB_NY + G	*n*-NY + G				

**Table 4:** Thermal properties of PEO, PCL, and Nylon 66 determined via DSC analysis. (*T*_g_: glass transition temperature; *T*_m_: melting temperature; Δ*H*_m_: melting enthalpy; χ_c_: degree of crystallization; *T*_c_: crystallization temperature).

Nanofibrous Mat	*T*_g_ (°C)	*T*_m_^*peak*^ (°C)	Δ*H*_m_ ^(a)^ (J/g)	χ_c_ (%)	*T*_c_^*onset*^ (°C)	*T*_c_^*peak*^ (°C)
*n*-PEO	n.d.	63	170	83.3	41	35
*n*-PEO + G	n.d.	64	177	86.8	44	39
*n*-PCL	−59.4	−63	78	55.9	32	25
*n*-PCL + G	−61.2	−61	79	55.6	39	34
*n*-NY	72.1	265	88	44.8	236	231
*n*-NY + G	64.6	266	87	44.4	247	239

^(a)^ In the case of G-reinforced nanofibers, the value is normalized on the actual polymer fraction. n.d. = not detectable.

**Table 5:** Mode I and Mode II delamination test results obtained via DCB and ENF tests, respectively.

Sample	Max Load in Mode I (N)	G_I,*C*_ (J/m^2^)	G_I,*R*_ (J/m^2^)	Max Load in Mode II (N)	G_II,*C*_ (J/m^2^)	G_II,*R*_ (J/m^2^)
DCB_Ref	37.1 ± 4.4	425 ± 111	350 ± 33	595 ± 6	1953 ± 178	2621 ± 448
DCB_PEO	63.6 ± 5.1	868 ± 143	1299 ± 210	646 ± 6	2400 ± 101	2945 ± 209
DCB_PEO + G	53.0 ± 2.6	537 ± 102	1098 ± 213	587 ± 34	1836 ± 87	2400 ± 261
DCB_PCL	52.5 ± 6.8	690 ± 142	781 ± 74	680 ± 28	2346 ± 157	2752 ± 288
DCB_PCL + G	32.3 ± 1.9	286 ± 40	331 ± 63	568 ± 12	1818 ± 176	2089 ± 86
DCB_NY	51.5 ± 3.2	699 ± 61	644 ± 76	623 ± 20	2036 ± 321	2606 ± 337
DCB_NY + G	30.0 ± 3.0	234 ± 39	249 ± 78	639 ± 18	2074 ± 248	2646 ± 348

## Data Availability

The data presented in this study are available on request from the corresponding author.
